# Topological bound states in the continuum in a non-Hermitian photonic system

**DOI:** 10.1515/nanoph-2024-0419

**Published:** 2025-01-14

**Authors:** Yihao Luo, Xiankai Sun

**Affiliations:** Department of Electronic Engineering, 26451 The Chinese University of Hong Kong, Shatin, New Territories, Hong Kong

**Keywords:** bound states in the continuum, topological photonics, non-Hermitian photonics, integrated photonics

## Abstract

Topological insulators and bound states in the continuum represent two fascinating topics in the optical and photonic domain. The exploration of their interconnection and potential applications has emerged as a current research focus. Here, we investigated non-Hermitian photonics based on a parallel cascaded-resonator system, where both direct and indirect coupling between adjacent resonators can be independently manipulated. We observed the emergence of topological Fabry−Pérot bound states in the continuum in this non-Hermitian system, and theoretically validated its robustness. We also observed topological phase transitions and exceptional points in the same system. By elucidating the relationship between topological insulators and bound states in the continuum, this work will enable various applications that harness the advantages of bound states in the continuum, exceptional points, and topology. These applications may include optical delay and storage, highly robust optical devices, high-sensitivity sensing, and chiral mode switching.

## 1 Introduction

Topological photonics is an emerging interdisciplinary field that draws inspiration from the concept of topological insulators in condensed matter physics [1], [2], [3], [4], [5]. It applies similar principles to manipulate the behavior of photons in specially designed structures, such as photonic crystals [6], [7], [8], [9], metamaterials [10], [11], and other artificially engineered structures [12], [13], [14], [15]. A key characteristic of topological photonics is the ability to create structures or materials that guide light along their boundaries or interfaces in a controlled and protected manner. These systems exhibit a high degree of robustness against defects and disorders, as they are insensitive to local disturbance [1], [2], [5]. The field of topological photonics shows great promise in various applications, including optical communication [16], robust sensing [12], and quantum information processing [17].

Conventionally, the description of physical systems relied on Hermitian Hamiltonians, which ensure the presence of real eigenvalues and the conservation of probabilities. However, the latter half of the twentieth century witnessed a significant shift as physicists delved into the exploration of non-Hermitian systems, characterized by Hamiltonians lacking Hermitian properties [18]. This exploration led to the discovery of PT-symmetric systems, where the symmetries of parity (P) and time-reversal (T) were found to preserve despite the absence of Hermiticity. In these systems, exceptional points (EPs) emerged as a crucial phenomenon, signifying degeneracies where eigenvalues and eigenvectors converge in the parameter space [19]. The unique attributes of EPs have found practical application in the designs of sensors, enabling highly sensitive and selective detection of minute perturbations [20]. Such EPs also hold great potential in chiral mode switching [21].

Bound states in the continuum (BICs) represent an interesting phenomenon where waves maintain perfectly localized despite coexisting with a continuous spectrum of radiation with energy dissipation [22]. The existence of BICs challenges conventional understanding and opens up intriguing possibilities, offering diverse applications in photonics [23], acoustics [24], integrated circuits [25], and plasmonics [26].

BICs also exhibit certain aspects of topological properties, which are characterized by topological charges in the wave vector space [27]. However, the correlation between the “band topology” of topological insulators and the “radiation topology” of BICs remains elusive [3]. To date, numerous studies have attempted to combine the concepts of topological insulators and BICs, which led to topological bound states in the continuum through astute band engineering [28], [29], [30]. Nevertheless, these existing topological bound states in the continuum rely primarily on Hermitian systems and lack explicit radiation channels, thereby imposing limitations on a comprehensive investigation of the physical nature and practical applications based on the relationship between the topological band theory and radiation theory.

Here, we proposed a non-Hermitian cascaded-resonator system with radiation channels on an integrated photonic platform. Through a rigorous theoretical analysis, we obtained topologically protected Fabry−Pérot bound states in the continuum (FP-BICs) in this non-Hermitian system by independently manipulating the direct and indirect coupling of the cascaded-resonator system. We also observed an evident topological phase transition and exceptional points in this system. To validate our theoretical findings, we conducted comprehensive numerical simulations with a finite-element method, confirming the existence of topological FP-BICs, which are consistent with our theoretical analysis. Compared with conventional FP-BICs, the proposed topological FP-BICs offer higher robustness and potential for enhanced localization due to the topological edge states, which can lead to higher *Q* factors and higher nonlinearity. Furthermore, conventional FP-BICs are affected by the direct coupling between resonators, which limits the shortest distance between adjacent resonators and makes it difficult to independently control the phase shifts and coupling strengths. The proposed topological FP-BICs overcome these limitations and feature greater compactness and tunability. Our research seamlessly integrates the realms of non-Hermitian systems, BICs, and topological insulators. The proposed non-Hermitian cascaded-resonator system holds immense potential for various applications that leverage the advantages of BICs, topology, and PT symmetry. These applications encompass optical delay, optical devices, high-sensitivity sensing, and chiral mode switching.

## 2 Theoretical analysis

**Figure 1:**
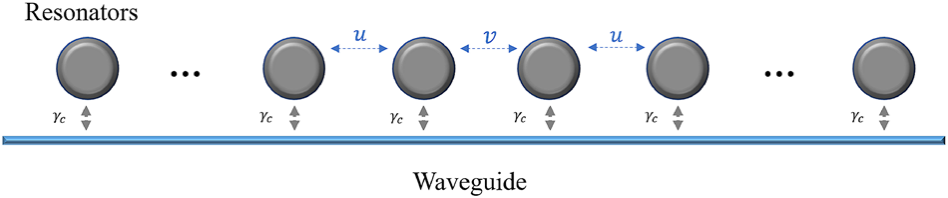
Schematic of the cascaded-resonator system with both direct and indirect coupling. The strengths of direct coupling between adjacent resonators are denoted as *u* and *v*. The indirect coupling between different resonators is achieved through the radiation channel in the bus waveguide, denoted as *γ*_c_.

Figure 1 illustrates our proposed cascaded-resonator system comprising an even number of resonators, which interact with each other with both direct and indirect coupling. In our model, we only consider the direct coupling between adjacent resonators, with the coupling strength alternating between *u* and *v*, and ignore other direct coupling from nonadjacent resonators. The indirect coupling between different resonators is achieved via a single-sided bus waveguide. We assume that all the resonators possess the same intrinsic frequency *ω*_0_, and the coupling strength between the resonators and the bus waveguide is *γ*_*c*_. Additionally, we assume that the phase shift between the adjacent resonators is *θ*. By applying the time-domain coupled-mode theory [31], we can express the Hamiltonian *H* of this system (see the Supplementary Information) as

(1) $$H=\left[\begin{array}{ccccccc} w_{0}-i\gamma_{c} & u-i\gamma_{c}{\text{e}}^{i\theta } & -i\gamma_{c}{\text{e}}^{i2\theta } & \ldots & \ldots & -i\gamma_{c}{\text{e}}^{i\left(N-2\right)\theta } & -i\gamma_{c}{\text{e}}^{i\left(N-1\right)\theta }\\ u-i\gamma_{c}{\text{e}}^{i\theta } & w_{0}-i\gamma_{c} & v-i\gamma_{c}{\text{e}}^{i\theta } & & \ldots & & -i\gamma_{c}{\text{e}}^{i\left(N-2\right)\theta }\\ -i\gamma_{c}{\text{e}}^{i2\theta } & v-i\gamma_{c}{\text{e}}^{i\theta } & w_{0}-i\gamma_{c} & u-i\gamma_{c}{\text{e}}^{i\theta } & & & \vdots \\ \vdots & \vdots & u-i\gamma_{c}{\text{e}}^{i\theta } & \ddots & \ddots & & \vdots \\ \vdots & \vdots & & \ddots & \ddots & v-i\gamma_{c}{\text{e}}^{i\theta } & \vdots \\ -i\gamma_{c}{\text{e}}^{i\left(N-2\right)\theta } & & & & v-i\gamma_{c}{\text{e}}^{i\theta } & w_{0}-i\gamma_{c} & u-i\gamma_{c}{\text{e}}^{i\theta }\\ -i\gamma_{c}{\text{e}}^{i\left(N-1\right)\theta } & -i\gamma_{c}{\text{e}}^{i\left(N-2\right)\theta } & \ldots & \ldots & -i\gamma_{c}{\text{e}}^{i2\theta } & u-i\gamma_{c}{\text{e}}^{i\theta } & w_{0}-i\gamma_{c} \end{array}\right].$$

The proposed system exhibits non-Hermiticity, which stems primarily from the radiation channel through the bus waveguide. It is evident that the diagonal terms of the Hamiltonian in Eq. (1) do not directly influence the band structure due to their uniformity. Consequently, we can decompose the system’s Hamiltonian into two components: *H* = *H*_eff_ + (*ω*_0_ − *iγ*_*c*_)*I*, where *I* is the identity matrix and *H*_eff_ is the Hamiltonian without the diagonal elements that ultimately determines the characteristics of the system’s band structure. Generally, the system does not satisfy PT symmetry or anti-PT (APT) symmetry. However, by carefully choosing the parameters, for example, *θ* = *π*/2 + *nπ* (*n* is an integer), the system satisfies PT symmetry, leading to the emergence of exceptional points. In this work, we only consider the case of an even number of resonators, because for a system with an odd number of resonators, PT symmetry can only be achieved under the condition of *u* = *v*, which precludes both the observation of EPs in the parameter space and the manipulation of the system’s topological properties. For the sake of simplicity, we consider a cascaded-resonator system comprising four resonators, whose effective Hamiltonian can be expressed as

(2) $$H_{\text{eff}}^{\left(4\right)}=\left[\begin{array}{cccc} 0 & u-i\gamma_{c}{\text{e}}^{i\theta } & -i\gamma_{c}{\text{e}}^{i2\theta } & -i\gamma_{c}{\text{e}}^{i3\theta }\\ u-i\gamma_{c}{\text{e}}^{i\theta } & 0 & v-i\gamma_{c}{\text{e}}^{i\theta } & -i\gamma_{c}{\text{e}}^{i2\theta }\\ -i\gamma_{c}{\text{e}}^{i2\theta } & v-i\gamma_{c}{\text{e}}^{i\theta } & 0 & u-i\gamma_{c}{\text{e}}^{i\theta }\\ -i\gamma_{c}{\text{e}}^{i3\theta } & -i\gamma_{c}{\text{e}}^{i2\theta } & u-i\gamma_{c}{\text{e}}^{i\theta } & 0 \end{array}\right].$$

**Figure 2:**
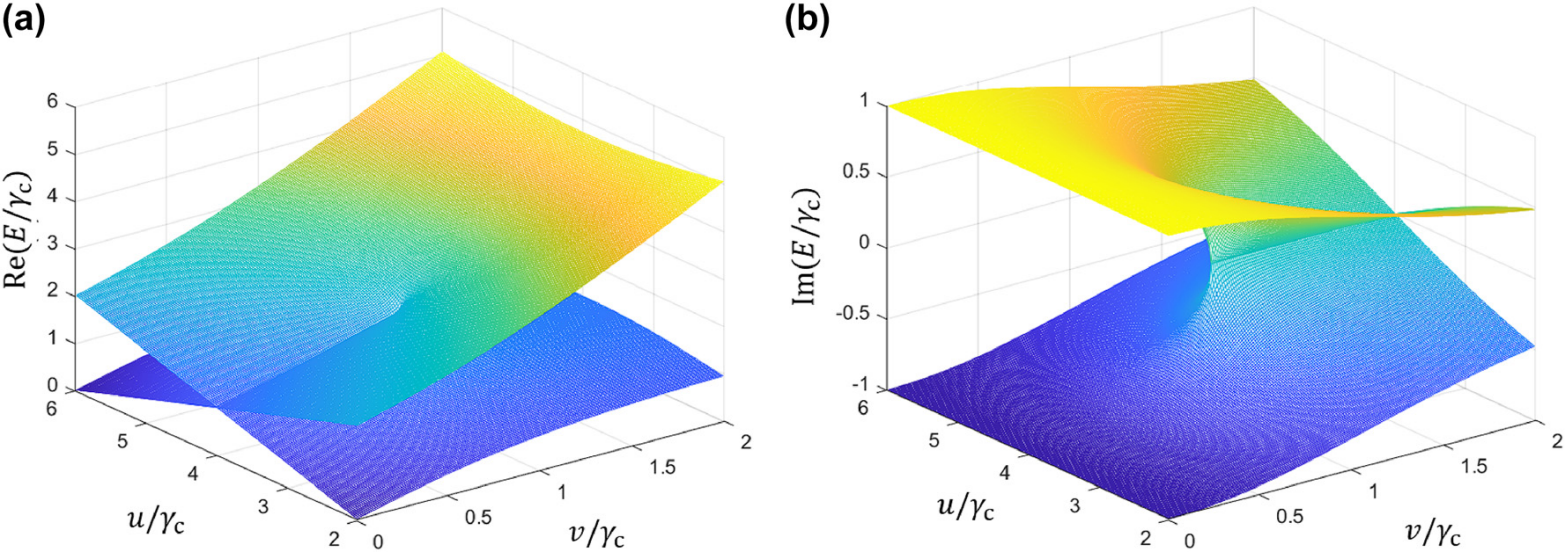
Real (a) and imaginary (b) part of the eigenvalues of a cascaded-resonator system comprising four resonators as a function of the direct coupling strengths *u* and *v*.

Through careful tuning of the parameters *u* and *v*, we observed EPs in the third and fourth energy bands of the system. Figure 2a and b depict the real and imaginary part of the energy eigenvalues, respectively. We can find that the EP appears at *u* = 4*γ*_*c*_ and *v* = *γ*_*c*_. Notably, if *θ* = *nπ*, one can achieve APT symmetry by modifying the resonator’s natural frequency, which has been discussed in Ref. [32].

**Figure 3:**
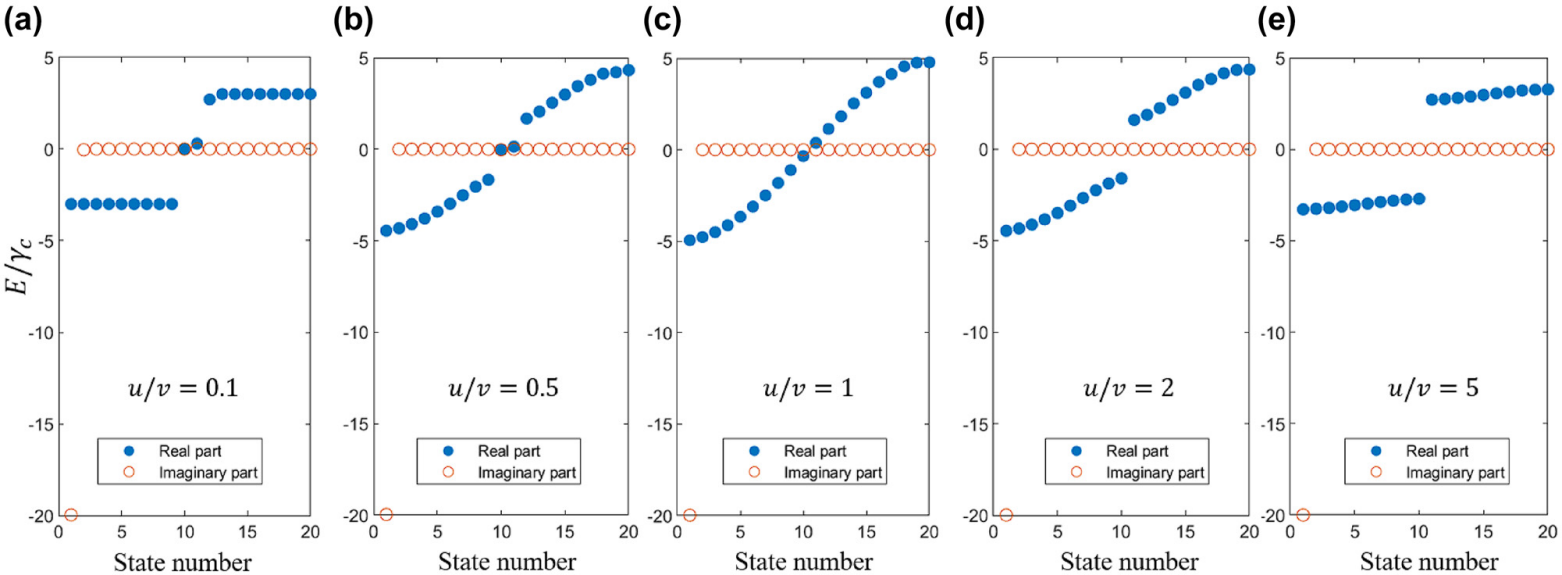
Eigenvalue distributions of a cascaded-resonator system comprising 20 resonators with *u*/*v* = 0.1 (a), 0.5 (b), 1 (c), 2 (d), 5 (e).

With *θ* = *nπ*, one can fulfill the coupling cancellation of different radiation channels to support FP-BICs in this system. First, let us consider the scenario where the direct coupling is absent (*u* = *v* = 0). In this case, the eigenvalues of the system can be solved analytically. In a cascaded-resonator system comprising *N* resonators, the eigenstates consist of (*N* − 1)-fold degenerate BICs, with the corresponding eigenvalue *ω*_0_, as well as a non-BIC lossy eigenstate, with the corresponding eigenvalue *ω*_0_ − *iNγ*_*c*_ [32]. When *u* and *v* are nonzero, it is challenging to obtain analytical solutions for the system’s eigenvalues. Therefore, we resort to numerical methods to analyze the eigenstates of the system. To investigate the influence of direct coupling on the distribution of eigenvalues, we calculated the eigenvalue distribution with varying *u*/*v* values, as depicted in Figure 3. For simplicity, we do not consider *ω*_0_ in the calculation, as it has no influence on the structure of the eigenvalue distributions.

Based on the numerical solutions, it is evident that the introduction of direct coupling significantly influences the distribution of the real part of eigenvalues for a finite system, but has negligible effect on their imaginary part so that the ideal BICs (with a zero imaginary part) turn into quasi-BICs (qBICs, with a negligibly small imaginary part). Further numerical analysis reveals that as the system size *N* increases to infinity, these qBICs transform into ideal BICs (see the Supplementary Information). Additionally, we observed that as *u*/*v* decreases, the bandgap undergoes a progressive closure followed by reopening, with two distinct eigenstates emerging in the gap when *u* < *v*. This intriguing behavior is closely associated with a topological phase transition.

**Figure 4:**
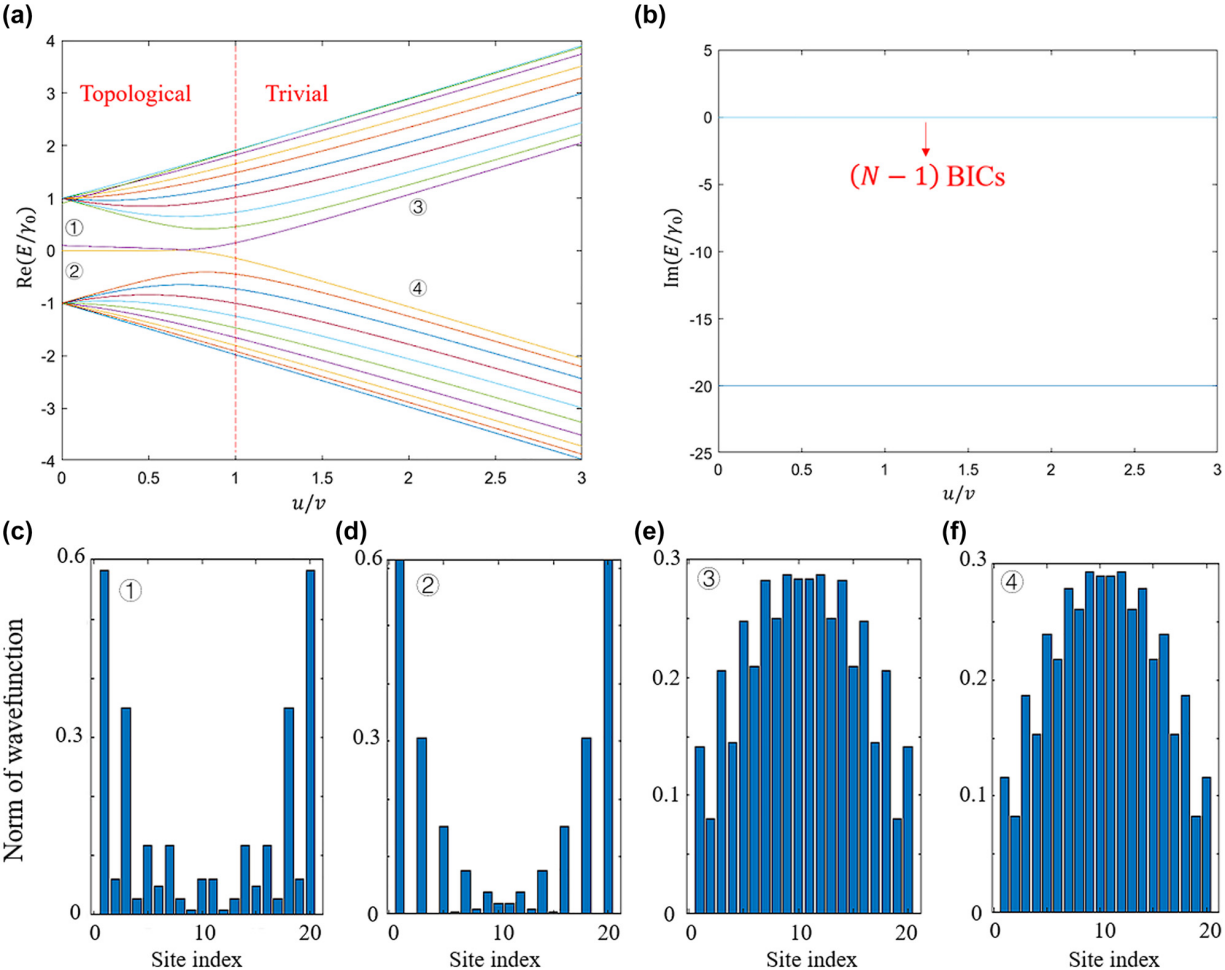
The energy spectrum and corresponding eigenstates, focusing on the case of *N* = 20 and *v* = *γ*_*c*_.(a), (b) Real (a) and imaginary (b) part of the eigenvalues of a cascaded-resonator system comprising 20 resonators as a function of *u*/*v*. (c)−(f) Norm of wavefunction of the system at the states ① to ④ marked in (a).

To investigate the topological phase transition in the system, we present the energy spectrum and corresponding eigenstates in Figure 4, focusing on the case of *N* = 20 and *v* = *γ*_*c*_. In Figure 4a, the real part of the energy eigenvalues reveals a distinct topological phase transition occurring at the critical point *u* = *v*. Specifically, when *u* < *v*, two topological edge states ① and ② emerge in the energy bandgap, whereas these edge states vanish when *u* > *v*. By contrast, in Figure 4b, the imaginary part of the energy eigenvalues remains largely unaffected by the introduction of direct coupling. Irrespective of the *u*/*v* values, the system consistently exhibits (*N* − 1) BICs and a lossy non-BIC state. Consequently, when *u* < *v* and *θ* = *nπ*, the system supports topological FP-BICs. The states labeled by ① and ② in Figure 4 correspond to the topological edge bound states in the continuum.

Indeed, for a system with periodic boundary conditions, its Hamiltonian *H* can be decomposed as a superposition of direct coupling *H*_*d*_, indirect coupling *H*_*i*_, and intrinsic resonance *ω*_0_, such that *H* = *H*_*d*_ + *H*_*i*_ + *ω*_0_, with

(3) $$H_{i}=\left[\begin{array}{cccc} \omega_{0}-i\gamma_{c} & -i\gamma_{c}{\text{e}}^{i\theta } & \ldots & -i\gamma_{c}{\text{e}}^{i\left(N-1\right)\theta }\\ -i\gamma_{c}{\text{e}}^{i\theta } & \omega_{0}-i\gamma_{c} & \ldots & -i\gamma_{c}{\text{e}}^{i\left(N-2\right)\theta }\\ \vdots & \vdots & \ddots & \vdots \\ -i\gamma_{c}{\text{e}}^{i\left(N-1\right)\theta } & -i\gamma_{c}{\text{e}}^{i\left(N-2\right)\theta } & \ldots & \omega_{0}-i\gamma_{c} \end{array}\right],$$

(4) $$H_{d}=\left[\begin{array}{cccccc} 0 & u & 0 & \ldots & 0 & v\\ u & 0 & v & \ddots & \ddots & 0\\ 0 & v & 0 & u & \ddots & \vdots \\ \vdots & \ddots & u & 0 & \ddots & 0\\ 0 & \ddots & \ddots & \ddots & \ddots & u\\ v & 0 & \ldots & 0 & u & 0 \end{array}\right].$$

This decomposition allows for a comprehensive analysis of the system’s topological properties. An intriguing observation arises when *θ* = *nπ*, as the components *H*_*d*_ and *H*_*i*_ commute, i.e., [*H*_*d*_, *H*_*i*_] = 0. Notably, *H*_*d*_ is identical to that from the Su–Schrieffer–Heeger (SSH) model, a Hermitian system with real eigenvalues. In this case, *u* is the intracell coupling and *v* is the intercell coupling. Conversely, *H*_*i*_ represents the non-Hermitian part of the system, which can be utilized to construct FP-BICs when *θ* = *nπ*. Despite the system’s non-Hermitian nature and intriguing features such as EPs, its topological properties are fundamentally inherited from the Hermitian part manipulated by direct coupling. By independently manipulating the direct and indirect coupling, it is possible to achieve topologically protected BICs in such a non-Hermitian system.

**Figure 5:**
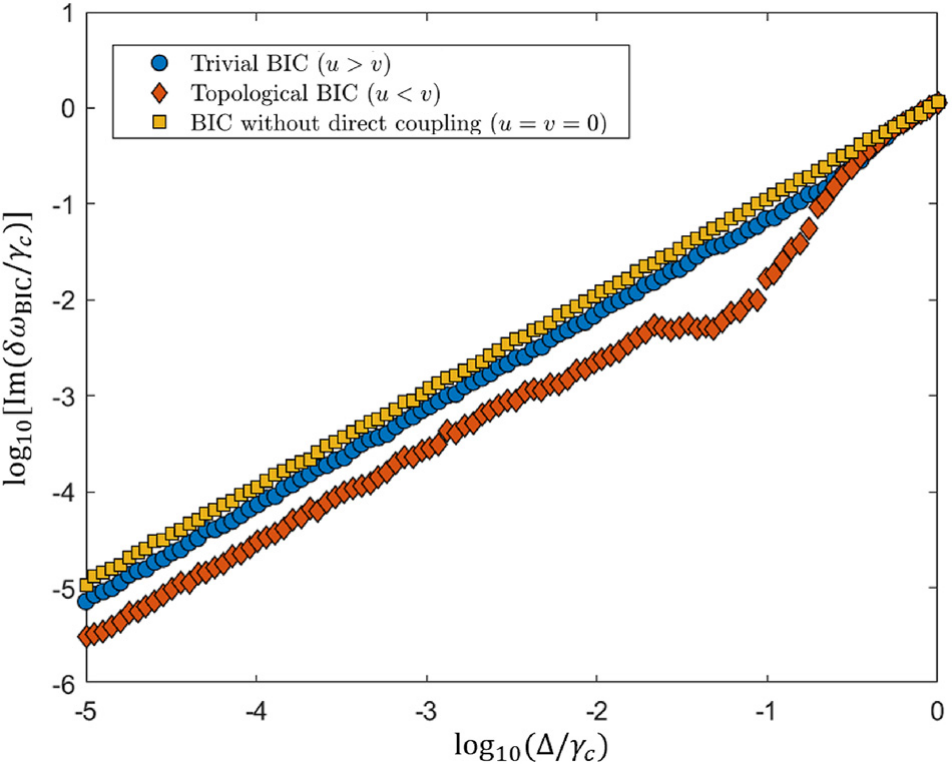
Robustness analysis of a cascaded-resonator system comprising 20 resonators.

To assess the system’s robustness, we introduced a random perturbation, denoted as *δH*, resulting in a modified Hamiltonian *H*′ = *H* + *δH*. Each element of *δH* satisfies the condition *δH*_*ij*_ ∈ (−Δ, Δ), where *δH*_*i,j*_ is the (*i,j*)th element of *δH* and Δ represents a non-negative parameter quantifying the perturbation strength. We generated *H*′ randomly 1,000 times by employing Δ of different magnitudes. Subsequently, we calculated the average variation of the imaginary part of the eigenfrequency Im(*δω*_BIC_) as an indicator of robustness. With the same Δ, a smaller Im(*δω*_BIC_) indicates a higher system robustness. Figure 5 shows the calculated results for three different scenarios. We calculated log_10_[Im(*δω*_BIC_/*γ*_*c*_)] at varying Δ when the system supports topological BICs (*u* < *v*), with the results shown by the orange diamonds in Figure 5. For comparison, we also calculated for the cases of trivial BICs (*u* > *v*) and BICs without direct coupling (*u* = *v* = 0), with the results shown by the blue circles and yellow squares in Figure 5 respectively. Our findings demonstrate that the introduction of topology enhances the robustness of BICs in the cascaded-resonator system.

## 3 Numerical simulation and application

**Figure 6:**
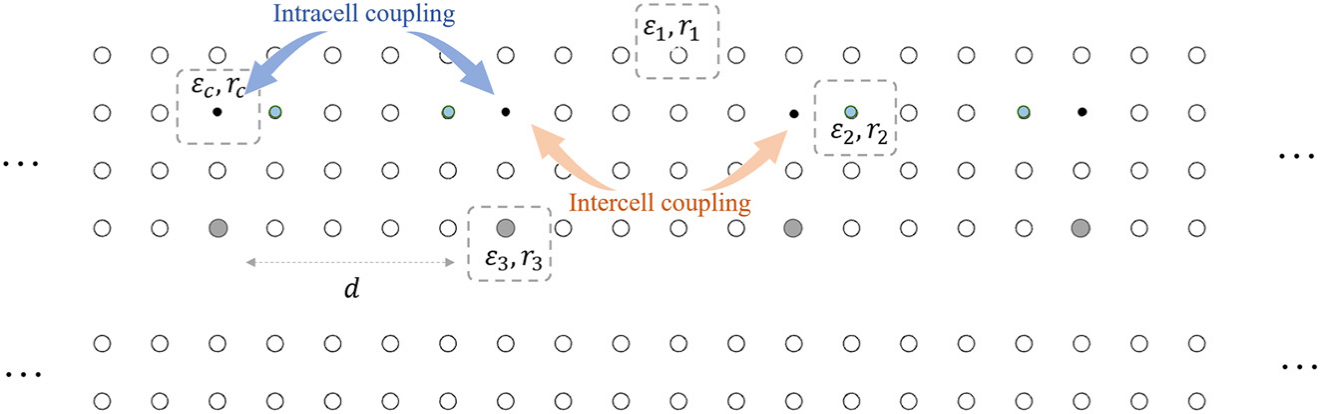
Implementation of the cascaded-resonator system on a two-dimensional photonic crystal platform.

To facilitate independent control of the direct coupling and indirect coupling, we proposed to employ two-dimensional photonic crystal platform for constructing the non-Hermitian cascaded-resonator system. Specifically, we introduce point defects in the photonic crystal to create resonators, and line defects to create waveguides. Figure 6 illustrates a possible implementation of such a cascaded-resonator system on a two-dimensional photonic crystal platform. The system comprises multiple dielectric cylinders with varying dielectric constants and radii. The photonic crystal is composed of cylinders with radius *r*_1_ and dielectric constant *ɛ*_1_, with lattice constant *a*. The point defects comprise cylinders with radius *r*_*c*_ and dielectric constant *ɛ*_*c*_. We modulate the direct coupling between adjacent resonators by using cylinders with radius *r*_2_ and dielectric constant *ɛ*_2_. In addition, we use another set of cylinders with radius *r*_3_ and dielectric constant *ɛ*_3_ to control the coupling between the resonators and the bus waveguide. There are 20 point defects in the system, with the same spacing *d* = 5*a* between adjacent point defects.

**Figure 7:**
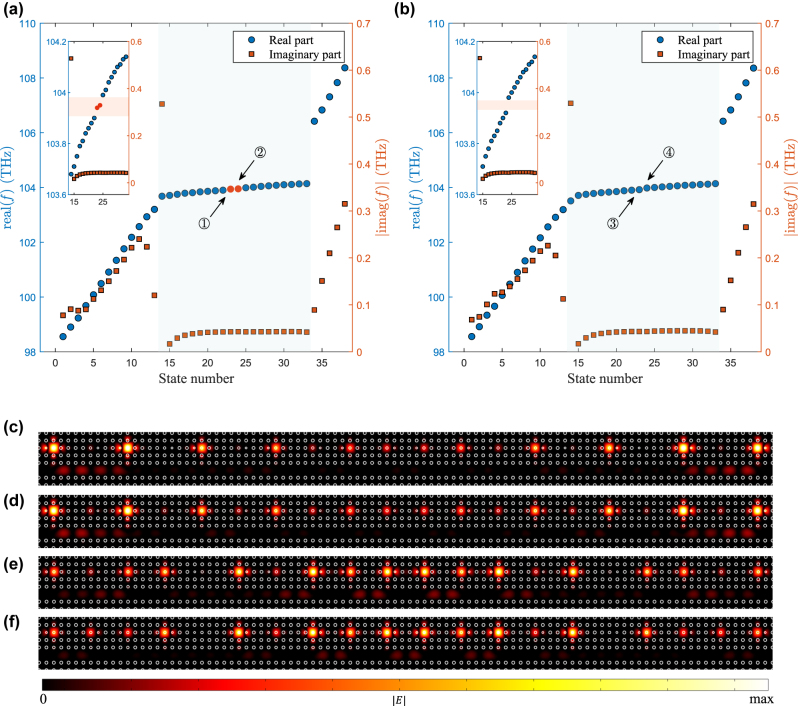
The complex eigenfrequencies of the system with scattering boundary conditions. (a), (b) Frequency spectra of a topological system (a) and a trivial system (b). The blue circles represent the real part of the complex frequency and the orange squares represent its imaginary part. The blue shaded regions mark the eigenstates of the cascaded-resonator system and the red shaded regions mark the bandgap. (c)–(f) Electric field distributions corresponding to the eigenstates denoted by ① – ④ in (a), (b).

With a finite-element method, we calculated the complex eigenfrequencies of the system with scattering boundary conditions. Figure 7a and b depict the complex eigenfrequencies under the condition of *u* < *v* and *u* > *v* respectively. The blue circles represent the real part of the complex eigenfrequencies, while the orange squares represent the imaginary part. The blue shaded regions mark the eigenstates of the cascaded-resonator system, while the unshaded regions mark the eigenstates of the waveguide. In the case of *u* < *v*, the system is topological, featuring two topological edge states in the bandgap, identified by red circles in Figure 7a. Conversely, when *u* > *v*, the system is trivial, displaying a bandgap without topological edge states, as illustrated in Figure 7b. Figure 7c–f present the electric field distributions corresponding to the eigenstates denoted by ① – ④ in Figure 7a and b, further verifying the presence of topological edge states. These findings verify the consistency between our simulations and theoretical analyses.

Our proposal for realizing topological Fabry–Pérot BICs in a non-Hermitian system not only establishes connections among topology, BICs, and PT symmetry but also opens up new opportunities and applications. First, the topological edge states in this non-Hermitian system are localized, offering advantages for realizing all-optical nonlinearity, including harmonic generation and four-wave mixing. These localized edge states possess dual advantages: (1) They are topologically protected, ensuring robustness against perturbations and imperfections. (2) They function as BICs with a high *Q* factor. These inherent properties make them particularly promising for applications in nonlinear optics. Additionally, by adjusting parameters such as the coupling coefficients (*u*, *v*) and the phase shift (*θ*) between adjacent resonators, one can realize dynamical encircling of an exceptional point. This feature can lead to intriguing chiral dynamics and provide opportunities for realizing asymmetric mode switching [33].

## 4 Conclusion and discussion

We proposed a non-Hermitian cascaded-resonator system that incorporates radiation channels on an integrated photonic platform for studying topological Fabry−Pérot BICs. Through a rigorous theoretical analysis, we obtained topologically protected FP-BICs in this non-Hermitian system by independently manipulating the direct and indirect coupling of the cascaded-resonator system. We also observed clear topological phase transitions and exceptional points in this system. We performed comprehensive numerical simulations with a finite-element method and confirmed the existence of topological FP-BICs, which agrees with our theoretical analysis. Our research seamlessly integrates the realms of non-Hermitian systems, bound states in the continuum, and topological insulators. The proposed non-Hermitian cascaded-resonator system exhibits significant potential for diverse applications that leverage the advantages of BICs, topology, and PT symmetry.

In conventional cascaded-resonator systems, such as cascaded microrings, one typically manipulates the direct and indirect coupling by tuning the spacing between individual resonators. In these scenarios, achieving independent control over both direct and indirect coupling proves challenging, as only one coupling channel remains accessible. Our proposed cascade-resonator system in this work has a notable advantage in its adjustability. In an implementation based on waveguide-coupled photonic crystal defect cavities, one can strategically design various point defects and carefully manipulate the spacing between resonators, to realize independent control over the interplay between each resonator and thus precisely regulate the strength of both direct and indirect coupling. By leveraging this system’s capabilities, we can realize optical delay lines, optical switches, filters, and other related applications through careful design. In addition, compared with microring resonators, our proposed system can be implemented on a more compact integrated photonic platform. We can also realize dynamical encircling of EPs in this system by altering the parameters of individual resonators, thereby broadening its potential for further advancements.

## Supplementary Material

Supplementary Material Details
