# Supplementary material for: Topological bound states in the continuum in a non-Hermitian photonic system
Source: Nanophotonics. 2025 Jan 14;14(1):43–50. doi: 10.1515/nanoph-2024-0419 (PMC11744451; doi:10.1515/nanoph-2024-0419)
Supplement: Supplementary file 1 — Supplementary Material Details [file j_nanoph-2024-0419_suppl_001.pdf]

Supplementary Information for "Topological bound states in the continuum in a non-Hermitian photonic system"

Yihao Luo and Xiankai Sun*

Department of Electronic Engineering, The Chinese University of Hong Kong, Shatin, New Territories, Hong Kong

*Corresponding author: xksun@cuhk.edu.hk

1 Derivation of the Hamiltonian of the cascaded-resonator system

In this section, we explain how Equation (1) in the main manuscript can be obtained. We can describe the electric field using temporal coupled-mode theory [S1] with the input-output relation for resonators:

$$\frac{d\phi_j}{dt} = -i\omega_j\phi_j - k_{j,j+1} - k_{j-1,j} - (\gamma_i + \gamma_c)\phi_j - \sqrt{\gamma_c}a^{(j)}_{+,\text{ in}}(t) - \sqrt{\gamma_c}a^{(j)}_{-,\text{ in}}(t), \tag{S1}$$

$$a^{(j+1)}_{+,\text{in}}(t) = e^{i\theta}a^{(j)}_{+,\text{out}}(t), \tag{S2}$$

$$a^{(j+1)}_{-,\text{out}}(t) = e^{-i\theta}a^{(j)}_{-,\text{in}}(t), \tag{S3}$$

$$a^{(j)}_{p,\text{ out}}(t) = a^{(j)}_{p,\text{ in}}(t) + \sqrt{\gamma_c}\phi_j, \tag{S4}$$

where ϕ_j is the annihilation operator associated with the mode of the jth ($j = 1, 2, \ldots, N$) resonator, with intrinsic resonant frequency ω_j. $a^{(j)}_{p,\text{ in}}$ is the input field coupled to the jth resonator, and $p = \pm$ represents the two propagating directions of the waveguide modes. γ_i and γ_c denote the intrinsic loss rates of all the resonators and the coupling rates between all the resonators and the bus waveguide. $k_{j,j+1}$ denote the coulping strength between the jth and $(j+1)$th resonator. Equation (S1)-(S4) can be simplified as

$$\frac{d\phi_1}{dt} = -i\omega_1\phi_1 - ik_{1,2} - (\gamma_c + \gamma_i)\phi_1 - \gamma_c\left(e^{i\theta}\phi_2 + e^{2i\theta}\phi_3 + \ldots + e^{(N-1)i\theta}\phi_N\right), \tag{S5}$$
$$-\sqrt{\gamma_c}a^{(1)}_{+,\text{in}}(t) - \sqrt{\gamma_c}e^{(j-1)i\theta}a^{(1)}_{-,\text{in}}(t),$$

$$\frac{d\phi_2}{dt} = -i\omega_2\phi_2 - ik_{1,2} - ik_{2,3} - (\gamma_c + \gamma_i)\phi_2 - \gamma_c\left(e^{i\theta}\phi_1 + e^{i\theta}\phi_3 + \ldots + e^{(N-2)i\theta}\phi_N\right) \tag{S6}$$
$$-\sqrt{\gamma_c}e^{i\theta}a^{(1)}_{+,\text{in}}(t) - \sqrt{\gamma_c}e^{(j-2)i\theta}a^{(1)}_{-,\text{in}}(t),$$

$$\cdots$$

$$\frac{d\phi_j}{dt} = -i\omega_j\phi_j - ik_{j-1,j} - (\gamma_c + \gamma_i)\phi_j - \gamma_c\left(e^{(j-1)i\theta}\phi_1 + e^{(j-2)i\theta}\phi_2 + \ldots + e^{i\theta}\phi_{j-1}\right. \tag{S7}$$
$$\left.+e^{i\theta}\phi_{j+1} + \ldots + e^{(N-j)i\theta}\phi_N\right) - \sqrt{\gamma_c}e^{(j-1)i\theta}a^{(1)}_{+,\text{in}}(t) - \sqrt{\gamma_c}a^{(1)}_{-,\text{in}}(t).$$

Therefore, the equation of motion for the cascaded-resonator system can be expressed as

$$i\partial_t\Psi = H\Psi + \mathcal{A}, \tag{S8}$$

where $\Psi = (\phi_1, \phi_2, \ldots, \phi_N)^T$ denotes the amplitudes of the N resonators, $\mathcal{A}$ is a driving term. We assumed $k_{2n-1,2n} = u$, $k_{2n,2n+1} = v$ (n is a positive integer), and $\omega_1 = \omega_2 = \omega_3 = \cdots = \omega_N = \omega_0$. The effective Hamiltonian can be expressed as

$$H = \begin{bmatrix} w_0 - i\gamma_c & u - i\gamma_c e^{i\theta} & -i\gamma_c e^{i2\theta} & \cdots & \cdots & -i\gamma_c e^{i(N-2)\theta} & -i\gamma_c e^{i(N-1)\theta} \\ u - i\gamma_c e^{i\theta} & w_0 - i\gamma_c & v - i\gamma_c e^{i\theta} & & \cdots & & -i\gamma_c e^{i(N-2)\theta} \\ -i\gamma_c e^{i2\theta} & v - i\gamma_c e^{i\theta} & w_0 - i\gamma_c & u - i\gamma_c e^{i\theta} & & & \vdots \\ \vdots & \vdots & u - i\gamma_c e^{i\theta} & \ddots & \ddots & & \vdots \\ \vdots & \vdots & & \ddots & \ddots & v - i\gamma_c e^{i\theta} & \vdots \\ -i\gamma_c e^{i(N-2)\theta} & & & & v - i\gamma_c e^{i\theta} & w_0 - i\gamma_c & u - i\gamma_c e^{i\theta} \\ -i\gamma_c e^{i(N-1)\theta} & -i\gamma_c e^{i(N-2)\theta} & \cdots & \cdots & -i\gamma_c e^{i2\theta} & u - i\gamma_c e^{i\theta} & w_0 - i\gamma_c \end{bmatrix}. \quad \text{(S9)}$$

Equation (S9) is exactly Equation (1) in the main manuscript.

2 Discussion on BICs and qBICs

In this section, we utilize numerical analysis to reveal that as the number of resonators N increases to infinity, the qBICs of the cascaded-resonator system will transform into ideal BICs. We calculate the imaginary part of the eigenvalues for qBICs under varying N. These results are illustrated in Figure S1. As the number of resonators increases, the average value of the imaginary part of the eigenvalue for qBIC gradually decreases to zero. It can be inferred that when the system size increases to infinity, there will be BICs without loss in the system.

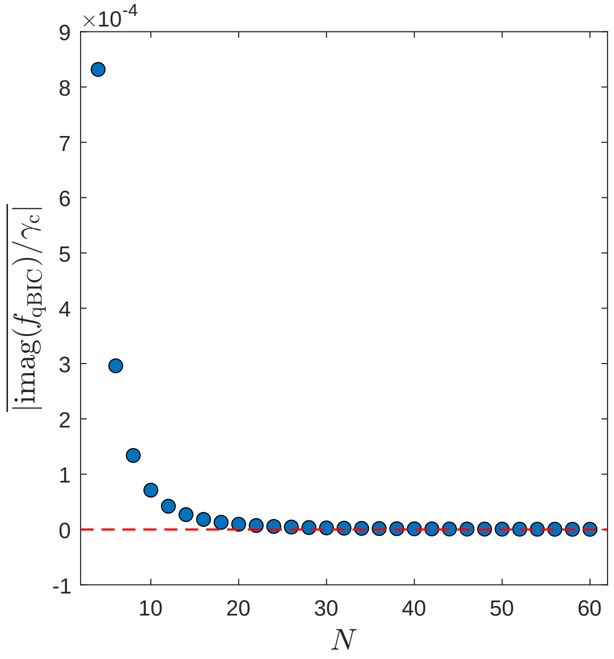

Figure S1. Average absolute value of the imaginary part of eigenvalues for qBIC as a function of N.

3 Discussion on the topological invariant of the system

Due to the nonlocality of the Hamiltonian, defining a topological invariant is challenging. However, by approximating the system as a "quasi-one-dimensional chain" while preserving its non-Hermitian properties, the winding number in the generalized Brillouin zone can be calculated using non-Bloch band theory. In this case, the Hamiltonian in the generalized momentum space can be defined as

$$H(\beta) = \begin{pmatrix} -i\gamma & u - v\beta^{-1} - i\gamma \\ u + v\beta - i\gamma & -i\gamma \end{pmatrix} \tag{S10}$$

where $\beta = e^{ik}$ is the general momentum. We further calculated the winding number of the system as depicted in Figure S2. When $u < v$, the winding number equals 1, which means that the system is topologically nontrivial. When $u > v$, the winding number equals 0, which means that the system is topologically trivial.

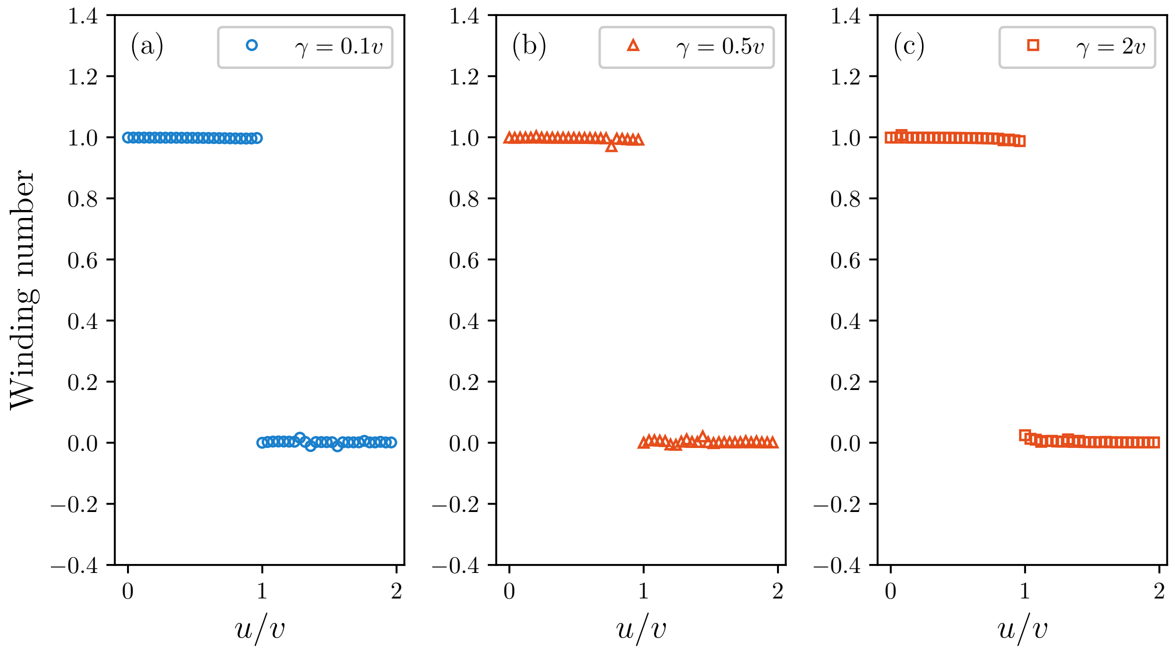

Figure S2. Winding number as a function of u/v with $\gamma = 0.1v$ (a), $\gamma = 0.5v$ (b), and $\gamma = 2v$ (c).

4 Simulation of the photonic crystal cascaded-resonator system

In this section, we present the parameter setting in the simulations of the photonic crystal cascaded-resonator system. As mentioned in the main manuscript, the spacing between the two adjacent resonators is $d = 5a$. To ensure radiation cancellation, which is requisite for FP–BIC, $kd = n\pi$ must be satisfied. This necessitates the careful tuning of a suitable transverse wave number k. With a finite–element method, we computed the dispersion relationship $k(\omega)$ of the waveguide, which is represented by the curve in the yellow shaded region of Figure S3. At $k = 0.6\pi/a$, the waveguide's eigenfrequency is 105 THz, and $kd = 2\pi$, which satisfy the radiation cancellation exactly.

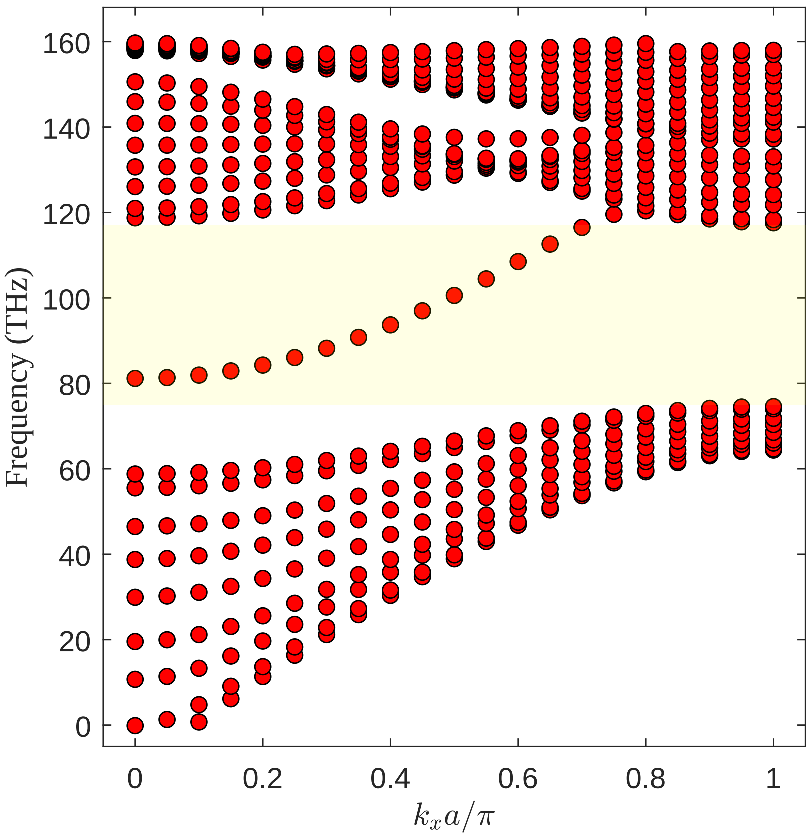

Figure S3. Eigenfrequency of the photonic crystal waveguide structure. The yellow shaded region marks the dispersion relationship of the waveguide.

To enable the coupling between resonators and the waveguide at $k = 0.6\pi/a$, careful tuning of ε_c and r_c are necessary to make the intrinsic frequency of the photonic crystal point defect $f_c = 105$ THz. We first set $\varepsilon_c = 11.56$, and calculated the eigenfrequency f_c of point defects under varying r_c. The results are shown in Figure S4. We find that when the eigenfrequency of the point defect is 105 THz, $r_c = 0.15$ μm, the coupling condition of the resonator and the waveguide at $k = 0.6\pi/a$ can be satisfied.

In addition to utilizing ε_c and r_c for tuning the eigenfrequency of resonators, we can further tune the direct coupling strength u and v between adjacent resonators by ε_2 and r_2. Similarly, the coupling strength between the resonators and the waveguide can be tuned by ε_3 and r_3 [S2, S3]. In simulations mentioned in the main manuscript, we set $\varepsilon_2 = 13.6$, $r_2 = 0.13$ μm, $\varepsilon_3 = 7.6$, and $r_2 = 0.18$ μm.

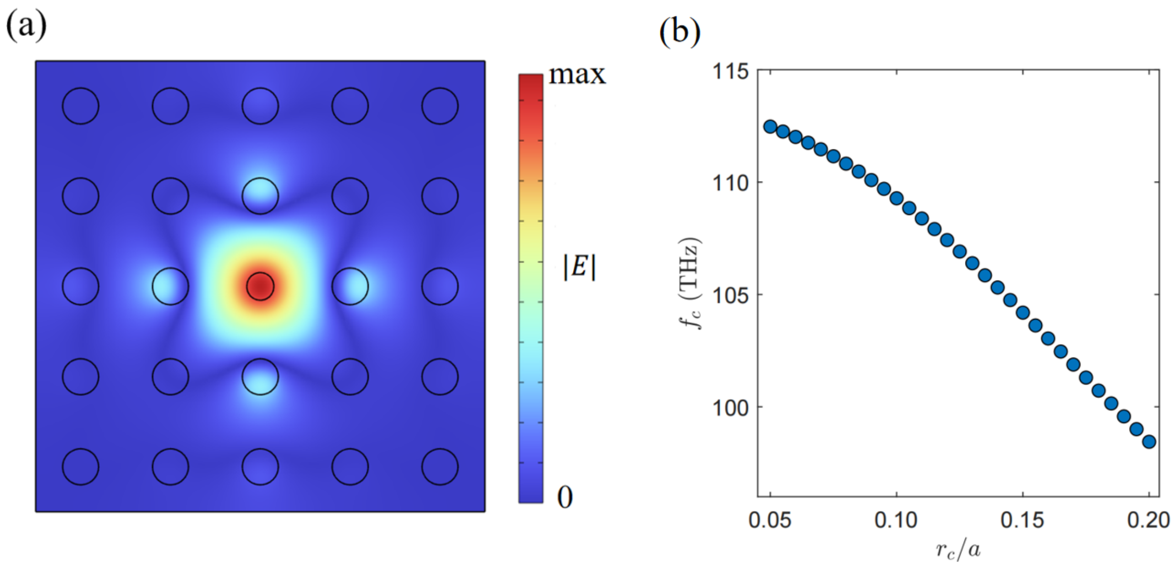

Figure S4. (a) Electric field distribution of the eigenstate of the photonic crystal point defect cavity. (b) Eigenfrequency of the point defect cavity as a function of r_c/a.

References

[S1] Xiao, Y., Li, M., Liu, Y., Li, Y., Sun, X., Gong, Q. Asymmetric Fano resonance analysis in indirectly coupled microresonators. *Phys. Rev. A* **82**, 065804 (2010).

[S2] Fan, S. et al. Theoretical analysis of channel drop tunneling processes. *Phys. Rev. B* **59**, 15882–15892 (1999).

[S3] Wang, Z. & Fan, S. Compact all-pass filters in photonic crystals as the building block for high-capacity optical delay lines. *Phys. Rev. E* **68**, 066616 (2003).
